# Creep Response of Carbon-Fiber-Reinforced Composite Using Homogenization Method

**DOI:** 10.3390/polym13060867

**Published:** 2021-03-11

**Authors:** Mostafa Katouzian, Sorin Vlase

**Affiliations:** 1 Department Machine Tools, Technical University of Munich, 85748 Munich, Germany; d-mec@unitbv.ro; 2 Department of Mechanical Engineering, Transilvania University of Brașov, B-dul Eroilor, 20, 500036 Brașov, Romania; 3 Romanian Academy of Technical Sciences, B-dul Dacia, 26, 030167 Bucharest, Romania

**Keywords:** homogenization, reinforced fiber composite, viscoelastic composite, carbon fiber, creep response, Young’s modulus, mechanical constants

## Abstract

The homogenization theory, used for the study of differential equations with periodic coefficients, with a rapid variation, is used in the paper for the analysis of the creep phenomenon of composite materials, reinforced with fibers. Generally, a polymer composite having a matrix with a viscoelastic response manifests a creep behavior. A good knowledge of mechanical constants allows us to predict the time response under the action of a load, which is important in engineering. The homogenization method is used to determine the engineering constants for a composite reinforced with carbon fibers. The method is applied for the particular case of fiber-reinforced unidirectional composites to obtain the equations that finally offer the required values. The epoxy matrix Fibredux 6376C is reinforced with carbon fibers T800 and the thermoplastic specimens made by APC2 material is reinforced with carbon fibers of the type IM6. The experimental results give a good concordance with the theoretical predictions.

## 1. Introduction

Creep phenomenon in viscoelastic materials represents a permanent deformation under a mechanical stress. This phenomenon can occur after a long-term exposure of the material to a high stress and is generally time-dependent. The rate of deformation depends on time exposure, temperature, level of stress and properties of the material [1,2]. Temperature is an important factor that increases significantly the rate of deformation. The material can increase in length and in some engineering application this can be undesirable. The creep behavior of the material can occur usually near the melting point, but for some materials this phenomenon can be manifested at the room temperature. Polymers, including plastics and reinforced plastics can reveal significant creep behavior even at room temperatures. The creep phenomenon in composite materials has been studied in detail by various authors, mainly due to the fact that composite materials are currently very widely used and because, in general, the materials used as matrices are visco-elastic.

The creep is characterized by creep strain, defined as the slope of the creep strain-time curve. In the design phase of a project, if the creep behavior occurs, it is necessary to know the rate of deformation. This is obtained by creep tests, when the creep strain–time curve is performed and can be implemented directly in the project [2,3]. A brief history of the domain and the basis of the creep studies are presented in [4,5,6].

Some results in the study of the creep phenomenon are presented below, to present the general framework of research in the field. In [7], an accelerated characterization scheme for the determination of the viscoelastic behavior of composites is proposed. This kind of approach leads to a reduced number of experimental measurements. Short-term observation done on a fiber reinforced composite at different temperatures allow the prediction of the long-term response. The studies use the time–temperature superposition principle (TTSP) [8,9,10].

Other studies concerning the nonlinear viscoelastic behavior of fiber reinforced composites has been made by Schaffer and Adams [11]. Their model uses a finite element micromechanics procedure, simplifying the analysis while using the symmetry pattern of such composite. The method can be applied not only for a bi-phasic material but also for a complex topology of a multiphasic composite.

Schapery [12,13] made a study of nonlinear viscoelastic materials, where thermal- and moisture-dilatational effects were considered through initial stresses. Mohan and Adams [14] applied this approach to study the graphite- and glass-reinforced composites and obtained a correlation between the method proposed by [11] and those proposed by [12,13].

Findley et al [15,16] proposed an empirical model to study the nonlinear creep compliance of a different composite, based on the creep power law. The method is well suited to numerical procedures. A nonlinear viscoelastic model using the Findley approach is applied to study of creep behavior of graphite/epoxy composites, by Dillard et al [17,18]. These theories were verified by measurements by Walrath [19].

An important stage in these analyses is to determine the constitutive law. These represents difficulties, as the material is inhomogeneous due to the reinforcing elements. The problem is to benefit from methods for calculating engineering constants which, for practical applications, must be simple to use. To achieve this goal, different averaging methods were utilized, using theorems from the mechanics of continuous media. Some results are summarized in the following.

Hashin et al, in series of investigations [20,21,22], used a method to pass from elastic to time-dependent behavior using a variational principle. It is assumed that the fiber volume ratio remain constant in different part of the composite despite the variation of diameters of fibers. A composite is considered to be a collection of cylindrical fibers with differing sizes. The assumption is that the strain energy of a representative element (a fiber surrounded by matrix) is equal to that of a homogeneous element.

Zhao et al [23] obtained results concerning the engineering constants which characterize an orthotropic and a transversely isotropic composite. Hill [24,25,26] determined the upper and lower bounds to estimate the elastic moduli of a fiber reinforced composite. Other researcher extended the Mori–Tanaka method [27,28] to study the viscoelastic response of an isotropic composite.

Significant results concerning the overall viscoelastic behavior of a composite reinforced with parallel fibers were obtained by Aboudi [29,30] in his micromechanical approach of one-dimensional composites. Another way to study this type of problem is that of the finite element analysis, with which a good agreement with the micromechanical model was obtained by Schaffer and Adams [11]. In other work, a micromechanical model to study the creep response of a composite carbon fiber reinforced composite is proposed [31]. To verify the model, experimental tests at different load and temperatures offer the creep curves.

The study of the mechanical properties of biphasic materials is made in numerous papers in the last few years [32,33,34,35,36,37,38,39], works that lead to the subject of the paper; namely, the use of the homogenization method for the calculation of elastic constants for such materials.

Over the last few decades, different mathematical estimation methods are used to determine the viscoelastic/elastic/plastic properties of a composite material. The mean field homogenization procedure is a precise method used in determining the mechanical properties of different types of composites, being able to analyze a wide class of materials, from unidirectional composites to textile composites, short fiber reinforced composites or nanotube-reinforced composites. The application of this optimization method for some practical cases is presented in [40], making a micro and meso-mechanical analysis of these types of materials. The estimation of the mechanical properties of the obtained material must be done using specific calculation methods. The homogenization method proves to be a very suitable method for solving this problem. In [41], this method is applied and the obtained results are compared with experimental results.

Other works refer to the theoretical aspect of the homogenization method, which involves the transition from a periodic structure of a composite material to a homogeneous structure [42]. Proposed analytical and numerical models have been used to determine the mechanical constants of a composite material. The obtained results were verified by the traction/compression and shear test. The experiments indicated that both obtained theoretical and numerical prediction values are in agreement with the results of experimental verifications confirming the validity of the methodology in providing a reliable reference for the structural design of pultruded fiber-reinforced polymer composite structures (FRP) [43].

A new method of homogenization of elasto-viscoplastic composites is developed in [44]. If Euler’s integration algorithm is used, the nonlinear ordinary differential equations in the mechanics of the elastic solid that describe the phase deformation of a composite can be discretized for a numerical approach. A total of three classical methods of approach can be used in this case: direct, secant and tangent. The interaction between the different phases of the composite material and the matrix in which these phases are incorporated is achieved by defining a new second-order tensor. In this way, the problems of homogenization of heterogeneous elasto-plastic and elasto-viscoplastic materials can be unified [45]. An example in which the engineering constants can be obtained analytically is presented and it is found that the results coincide with the values known in the literature [46]. During the research, experimental measurements are performed to verify the computed values. It is found that, for most of the tests performed for the specimens of carbon fiber reinforced composites, the results are very close to the experimentally determined values.

The research carried out at present deals mainly with the refinement of the proposed methods and with applications to different situations that may be encountered in practice [47,48,49,50,51,52,53,54]. Related methods of studying the problem are presented in [55,56]. Interest in the application of the theory of homogenization for this type of problem exists, but specific models for the application of homogenization are rarely found in the literature. The development of models with experimentally verified results is useful for researchers and manufacturers of such composites.

In the paper, the homogenization theory previously presented is applied to obtain the mechanical constants of a composite material. Thus, the general theory is applied for the special case of fiber-reinforced unidirectional composites. The equations are obtained that finally offer the required values. In general, obtaining these values involves numerical procedures, which was applied for the materials studied in the paper.

## 2. Homogenized Model of Carbon Fiber Composite

Generally, in mathematics, homogenization represents a method for the study of partial differential equations having rapidly oscillating coefficients. This becomes important for some types of continuous materials such composite materials with a periodical structure. In this case the coefficients of differential equations describing the response of material a periodical. Such material is convenient to treat as homogeneous, despite the fact that the material possess a particular microstructure. The homogenization procedure replaces an equation having high oscillatory coefficients with an equation with constant coefficients and represents an extension of the continuum concept to a new class of materials: materials with microstructure. The foundations of homogenization method are presented in a series of papers, i.e., [57,58,59,60,61,62]. In the following, this theory is applied for a fiber reinforced composite.

### 2.1. Macroscopic Equations

The stress field $\sigma^{\delta }$ for the case when the entire material is subdivided into repeating unit cells of dimension δ is:

(1) $$\frac{\partial \sigma_{11}^{\delta }}{\partial x_{1}}+\frac{\partial \tau_{12}^{\delta }}{\partial x_{2}}+\frac{\partial \tau_{13}^{\delta }}{\partial x_{3}}=f_{1}(x)\;\frac{\partial \tau_{21}^{\delta }}{\partial x_{1}}+\frac{\partial \sigma_{22}^{\delta }}{\partial x_{2}}+\frac{\partial \tau_{23}^{\delta }}{\partial x_{3}}=f_{2}(x)\;\frac{\partial \tau_{31}^{\delta }}{\partial x_{1}}+\frac{\partial \tau_{32}^{\delta }}{\partial x_{2}}+\frac{\partial \sigma_{33}^{\delta }}{\partial x_{3}}=f_{3}(x)$$

with $\sigma_{ij}^{\delta }=\sigma_{ji}^{\delta }$, for i,j=1,2,3.

The field of displacements $u^{\delta }$ should satisfy the contour conditions:

(2) $${\left.u^{\delta }\right|}_{\partial_{1}\Omega }=\tilde{u}$$

on the other hand, the stresses should satisfy the boundary conditions:

(3) $$\sigma_{11}^{\delta }n_{1}+\tau_{12}^{\delta }n_{2}+\tau_{13}^{\delta }n_{3}=T_{1}(x)\;\tau_{21}^{\delta }n_{1}+\sigma_{22}^{\delta }n_{2}+\tau_{23}^{\delta }n_{3}=T_{2}(x)\;\tau_{31}^{\delta }n_{1}+\tau_{32}^{\delta }n_{2}+\sigma_{33}^{\delta }n_{3}=T_{3}(x)$$

on the contour $\partial_{2}\Omega$,($\partial_{1}\Omega \cup \partial_{2}\Omega =\partial \Omega$). The stress–strain relation in the local domain can be written according to the Hook’s Law as:

(4) $${\left\{\begin{array}{c} \sigma_{11}\\ \sigma_{22}\\ \sigma_{33}\\ \tau_{23}\\ \tau_{31}\\ \tau_{12} \end{array}\right\}}^{\delta }=\left[\begin{array}{cccccc} C_{1111} & C_{1122} & C_{1133} & & & \\ C_{2211} & C_{2222} & C_{2233} & & 0 & \\ C_{3311} & C_{3322} & C_{3333} & & & \\ & & & C_{2323} & & \\ & 0 & & & C_{3131} & \\ & & & & & C_{1212} \end{array}\right]{\left\{\begin{array}{c} \varepsilon_{11}\\ \varepsilon_{22}\\ \varepsilon_{33}\\ \gamma_{23}\\ \gamma_{31}\\ \gamma_{13} \end{array}\right\}}^{\delta }$$

or in the compact form as:

(5) $$\sigma^{\delta }=C\varepsilon^{\delta }$$

The above elasticity matrix is semi-positive definite with coefficient $C_{ijkh}$ being functions of *x*. One can write:

(6) $$C_{ijkh}x_{ij}x_{kh}\geq \alpha \;x_{ij}x_{kh}$$

for α>0 and $\forall x_{ij},x_{kh}\in R$. In the analysis of composite materials, $C_{ijkh}(x)$ are periodical function of *x*, with the period having the dimension δ of the unit cell. Having introduced the notation y=x/δ, these coefficients become a function of *y* in domain Γ of the unit periodic cell:

(7) $$C_{ijkh}(x)=C_{ijkh}(y\delta )=C_{ijkh}(y)$$

Let us illustrate the applicability of the above system of equations by introducing a model which can be used to obtain the solution of Equation (1) written earlier. Let further the stress field in the unit cell be expressed in the following form:

(8) $$\sigma_{ij}^{\delta }=\sigma_{ij}^{o}(x,y)+\sigma_{ij}^{1}(x,y)\delta +.....$$

Note that the dependence of stress on *y* is “quasi-periodical”.

By applying Equation (8) to the equilibrium Equation (1) it follows that:

(9) $$\delta^{-1}\frac{\partial \sigma_{ij}^{o}}{\partial y_{j}}+\left(\frac{\partial \sigma_{ij}^{o}}{\partial x_{j}}+\frac{\partial \sigma_{ij}^{1}}{\partial y_{j}}\right)\;\delta^{o}+\left(\frac{\partial \sigma_{ij}^{1}}{\partial x_{j}}+\frac{\partial \sigma_{ij}^{2}}{\partial y_{j}}\right)\;\delta^{1}+.....=f_{i}(x)$$

Note that in the derivation of the above equation, use is made of the following property. For a function *f* which depends on *x* and *y* where *y* itself is a function of *x*, one can write:

(10) $$\frac{d}{dx}\left(f\right)=\frac{\partial f}{\partial x}dx+\frac{\partial f}{\partial x}dy$$

but since y=x/δ and thus dy=dx/δ; therefore:

(11) $$\frac{d}{dx}\left(f\right)=\frac{\partial f}{\partial x}\left(f\right)+\frac{1}{\delta }\frac{\partial }{\partial y}\left(f\right)$$

Identification of the terms with the coefficients of $\delta^{-1}$ in Equation (9) yields:

(12) $$\frac{\partial \sigma_{ij}^{o}}{\partial y_{j}}=0$$

which is called the “local equation”. In this equation, $\sigma_{ij}^{o}$ is a function of *y* and *x*. However, since the dependence of $\sigma_{ij}^{o}$ on *x* is very weak, *x* can be taken as a parameter, constant in the domain Γ. On the other hand, the function $\sigma_{ij}^{o}$ depends on *y* in a periodical way. This in turn means that one can consider this equation only within the domain of a cell Γ. By solving this differential system “the homogenized elastic matrix” can be established.

Similarly, if one identifies the corresponding terms of $\delta^{o}$, following equations are obtained:

(13) $$\frac{\partial \sigma_{ij}^{o}}{\partial x_{j}}+\frac{\partial \sigma_{ij}^{1}}{\partial y_{j}}=f_{i}(x)\;i=1,2,3$$

These are referred to as “microscopic equations” and they contain both variations on *x* as well as on *y*. By applying the average operator to this equation, it follows that:

(14) $$\frac{\partial \langle \sigma_{ij}^{o}\rangle }{\partial x_{j}}+\langle \frac{\partial \sigma_{ij}^{1}}{\partial y_{j}}\rangle =f_{i}(x)\;i=1,2,3$$

The average operator represents the average of the considered size over the domain *V*. The main idea here is to “average” the variation on y which is considered to be very small in comparison with the variation on x in the domain Γ. On the other hand:

(15) $$\langle \frac{\partial \sigma_{ij}^{1}}{\partial y_{j}}\rangle =\frac{1}{V}\int_{V}\partial \sigma_{ij}^{1}dV=\frac{1}{V}\int_{\partial V}\sigma_{ij}^{1}n_{j}dS=0$$

Note that $\sigma_{ij}^{1}$ take equal values on the corresponding points of the boundary of the cell Γ (following from the property of periodicity). At the same points, $n_{j}$ takes opposite values as a results of which:

(16) $$\frac{\partial \langle \sigma_{ij}^{o}\rangle }{\partial x_{j}}=f_{i}(x)\;i=1,2,3$$

By integration of this equation, the homogenized displacement field $u^{o}$ is obtained for the entire domain Ω.

### 2.2. Evaluation of the Homogenized Coefficients

In order to evaluate the homogenized coefficients that are functions of the microstructure in a heterogeneous medium, following notations are introduced:

(17) $$\varepsilon_{ij,x}(w)=\frac{1}{2}\left(\frac{\partial w_{i}}{\partial x_{j}}+\frac{\partial w_{j}}{\partial x_{i}}\right)\;;\;i,j=1,2,3$$

(18) $$\varepsilon_{ij,y}(w)=\frac{1}{2}\left(\frac{\partial w_{i}}{\partial y_{j}}+\frac{\partial w_{j}}{\partial y_{i}}\right)\;;\;i,j=1,2,3$$

Let the displacement field be approximated by:

(19) $$u(x,y)=u^{o}(x)+u^{1}(x,y)\;\delta +u^{2}(x,y)\;\delta^{2}+....$$

In this equation $u^{o}(x)$ depends only on *x* and terms $u^{1}(x,y)\;\delta$ and $u^{2}(x,y)$ are taken as quasi-periodical. Now using the above notations, one may write:

(20) $$\varepsilon_{kh,x}(u)=\frac{1}{2}\left(\frac{\partial u_{k}}{\partial x_{h}}+\frac{\partial u_{h}}{\partial x_{k}}\right)\;\;=\frac{1}{2}\left(\frac{\partial u_{k}^{o}}{\partial x_{h}}+\frac{\partial u_{h}^{o}}{\partial x_{k}}\right)+\frac{\delta }{2}\left(\frac{\partial u_{k}^{1}}{\partial x_{h}}+\frac{\partial u_{h}^{1}}{\partial x_{k}}\right)+\frac{\delta }{2}\left(\frac{\partial u_{k}^{2}}{\partial y_{h}}+\frac{\partial u_{h}^{2}}{\partial y_{k}}\right)+....\;\;=\varepsilon_{kh,x}(u^{o})+\varepsilon_{kh,y}(u^{1})+\delta \left[\varepsilon_{kh,x}(u^{1})+\varepsilon_{kh,y}(u^{2})\right]+\delta^{2}\left[\ldots \right].+\ldots \;;\;k,h=1,2,3$$

which can be simplified to:

(21) $$\varepsilon_{kh,x}(u)=\varepsilon_{kh}^{o}+\delta \;\varepsilon_{kh}^{1}+\ldots \;;\;k,h=1,2,3$$

where:

(22) $$\varepsilon_{kh}^{o}=\varepsilon_{kh,x}(u^{o})+\varepsilon_{kh,y}(u^{1})\;k,h=1,2,3$$

(23) $$\varepsilon_{kh}^{1}=\left[\varepsilon_{kh,x}(u^{1})+\varepsilon_{kh,y}(u^{2})\right]\;;\;k,h=1,2,3$$

In other words, the infinitesimal term $u^{1}(x,y)\delta$ represents a finite component of $\varepsilon_{kh}$, the term that should be taken into account when applying the Hooke’s law:

(24) $$\sigma_{ij}^{o}=C_{ijkh}\varepsilon_{kh}^{o}\;,\;i,j,k,h=1,2,3$$

From the local equation in (24), it follows that:

(25) $$\frac{\partial \left(C_{ijkh}\varepsilon_{kh}^{o}\right)}{\partial y_{j}}=0\;,\;i,j,k,h=1,2,3$$

or:

(26) $$\frac{\partial \left[C_{ijkh}\left(\varepsilon_{kh,x}^{}(u^{o})+\varepsilon_{kh,y}^{}(u^{1})\right)\right]}{\partial y_{j}}=0\;,\;i,j,k,h=1,2,3$$

The terms $\varepsilon_{kh,x}^{}(u^{o})$ depend only on *x* and thus may be considered constant for the current problem. The last of the above equations is therefore written in the alternative form as:

(27) $$-\frac{\partial \left[C_{ijkh}\;\varepsilon_{kh,y}^{}(u^{1})\right]}{\partial y_{j}}=\varepsilon_{kh,x}(u^{o})\frac{\partial C_{ijkh}}{\partial y_{j}}\;,\;i,j,k,h=1,2,3$$

By substituting:

(28) $$u^{1}=w^{kh}\varepsilon_{kh,x}^{}(u^{o})+k(x)$$

In Equation (27) with *k*(*x*) being an arbitrary function on *x*, it can be shown that:

(29) $$\varepsilon_{lm,y}^{}(u^{1})=\varepsilon_{kh,x}^{}(u^{o})\frac{1}{2}\left(\frac{\partial w_{l}^{kh}}{\partial y_{m}}+\frac{\partial w_{m}^{kh}}{\partial y_{l}}\right)=\varepsilon_{kh,x}^{}(u^{o})\varepsilon_{lm,y}^{}(w^{kh})$$

Therefore, the differential Equation (29) becomes:

(30) $$-\varepsilon_{kh,x}^{}(u^{o})\frac{\partial \left[C_{ijlm}\varepsilon_{lm,y}(w^{kh})\right]}{\partial y_{j}}=\varepsilon_{kh,x}(u^{o})\frac{\partial C_{ijkh}}{\partial y_{j}}\;,\;i,j,k,h=1,2,3$$

This relation should remain valid for any strain field $\varepsilon_{kh,x}(u^{o})$. Equation (30) can be simplified to:

(31) $$-\frac{\partial \left[C_{ijlm}\varepsilon_{lm,y}(w^{kh})\right]}{\partial y_{j}}=\frac{\partial C_{ijkh}}{\partial y_{j}}\;,\;i,j,k,h=1,2,3$$

Let us now make use of the relations:

(32) $$\int_{\Gamma }\frac{\partial \left[C_{ijlm}\varepsilon_{lm,y}(w^{kh})\right]}{\partial y_{j}}v_{i}dV+\int_{\Gamma }C_{ijlm}\varepsilon_{lm,y}(w^{kh})\frac{\partial v_{i}}{\partial y_{j}}dV=\int_{\partial \Gamma }u_{i}C_{ijlm}\varepsilon_{lm,y}(w^{kh})v_{i}^{}dS=0\;,\;i,j,k,h=1,2,3$$

(33) $$\int_{\Gamma }\frac{\partial \left[C_{ijlm}\varepsilon_{lm,y}(w^{kh})\right]}{\partial y_{i}}v_{j}dV+\int_{\Gamma }C_{ijlm}\varepsilon_{lm,y}(w^{kh})\frac{\partial v_{j}}{\partial y_{i}}dV=\int_{\partial \Gamma }u_{j}C_{ijlm}\varepsilon_{lm,y}(w^{kh})v_{j}dS=0\;,\;i,j,k,h=1,2,3$$

Note that in the second equation, the indices *i* and *j* have been interchanged and the property $C_{ijlm}=C_{jilm}$ has been used. It should be mentioned that here the Green’s theorem was applied while making use of the property that the functions $u_{i},C_{ijlm},\varepsilon_{lm}$ and $v_{i}$ are Y-periodical. From these two relations, one can write:

(34) $$\int_{\Gamma }\frac{\partial \left[C_{ijlm}\varepsilon_{lm,y}(w^{kh})\right]}{\partial y_{j}}v_{i}dV+\int_{\Gamma }\frac{\partial \left[C_{ijlm}\varepsilon_{lm,y}(w^{kh})\right]}{\partial y_{i}}v_{j}dV\;=2\int_{\Gamma }C_{ijlm}\varepsilon_{lm,y}(w^{kh})\frac{1}{2}\left(\frac{\partial v_{i}}{\partial y_{j}}+\frac{\partial v_{j}}{\partial y_{i}}\right)dV=2\int_{\Gamma }C_{ijlm}\varepsilon_{ij,y}(v)\varepsilon_{lm,y}(w^{kh})dV\;,\;i,j,k,h=1,2,3$$

Now, multiplying both sides of Equation (34) by v while taking into consideration the property $C_{ijlm}=C_{jilm}$, it follows that:

(35) $$\frac{\partial \left[C_{ijlm}\varepsilon_{lm,y}(w^{kh})\right]}{\partial y_{j}}v_{i}=\frac{\partial C_{ijkh}}{\partial y_{j}}v_{i}.$$

Interchanging the indices *i* and *j* yields:

(36) $$\frac{\partial \left[C_{ijlm}\varepsilon_{lm,y}(w^{kh})\right]}{\partial y_{i}}v_{j}=\frac{\partial C_{ijkh}}{\partial y_{i}}v_{j}$$

Integration and addition of the above two relations, while making use of the Equation (34) leads to:

(37) $$\int_{\Gamma }C_{ijlm}\varepsilon_{ij,y}(v)\varepsilon_{lm,y}(w^{kh})dV=\int_{\Gamma }\frac{\partial C_{ijkh}}{\partial y_{j}}v_{i}dV$$

In variational formulation, the problem is now to find $w^{kh}$ in $V_{y}$ such that $\forall v\in V_{y}$, the previous relation holds. When $w^{kh}$ is determined, then:

(38) $$\sigma_{ij}^{o}=C_{ijkh}\left[\varepsilon_{kh,x}^{}(u^{o})+\varepsilon_{kh,y}^{}(u^{1})\right]\;=C_{ijkh}\left[\varepsilon_{kh,x}^{}(u^{o})+\varepsilon_{kh,x}^{}(u^{o})\;\varepsilon_{lm,y}\left(w^{kh}\right)\right]$$

By applying the average operator, it follows that:

(39) $$\langle \sigma_{ij}^{o}\rangle =\langle C_{ijkh}\varepsilon_{kh,x}(u^{o})\rangle +\langle C_{ijkh}\varepsilon_{kh,x}(u^{o})\varepsilon_{lm,y}(u^{kh})\rangle \;=\langle C_{ijkh}\rangle \varepsilon_{kh,x}(u^{o})+\langle C_{ijkh}\varepsilon_{lm,y}(u^{kh})\rangle \varepsilon_{kh,x}(u^{o})$$

Furthermore:

(40) $$\langle \sigma_{ij}^{o}\rangle =\left[\langle C_{ijkh}\rangle +\langle C_{ijkh}\varepsilon_{lm,y}(u^{kh})\rangle \right]\varepsilon_{kh,x}(u^{o})$$

Comparison of this equation with:

(41) $$\langle \sigma_{ij}^{o}\rangle =C_{ijkh}^{o}{\tilde{\varepsilon }}_{kh}(u^{o})$$

while making use of the notation $\varepsilon_{kh,x}(u^{o})\equiv {\tilde{\varepsilon }}_{kh}(u^{o})$, yields the homogenized coefficients:

(42) $$C_{ijkh}^{o}=\langle C_{ijkh}\rangle +\langle C_{ijkh}\varepsilon_{lm,y}(u^{kh})\rangle$$

This means that in order to determine the homogenized coefficient, one has to calculate the Y-periodic function $w^{kh}$ with $\langle w^{kh}\rangle =0$ which verifies the differential Equation (30).

In summary, there are two ways to evaluate the homogenized coefficients:

- If one starts from local equations, it is possible to determine the strain and stress field. By using the averages, the homogenized coefficients can be evaluated;
- One can use the variational formulation and find the function $w^{kh}$ which allows computation of the homogenized coefficients.

For illustrative purpose, the above theory is applied to calculate the effective Young’s modulus of a composite in a one dimensional case (Appendix A).

### 2.3. Evaluation of Homogenized Coefficients for FRP

In the case of a fiber reinforced composite, there are two distinct phases present. These phases are the fiber and matrix materials denoted by *f* and *m,* respectively. Let $v_{f}=V_{f}/V$ and $v_{m}=V_{m}/V$ represent the volume fractions of the fiber and matrix in the composite where $V=V_{f}+V_{m}$ is the total volume of the representative unit cell. Due to the existing regular and periodic pattern of the fiber packing, it seems appropriate to limit the analysis to a repeating unit cell. Conforming to the previously described theory, there exists a class of solution $w^{kh}$, with *k,h* = 1,2,3 which satisfies the differential equations:

(43) $$-\frac{\partial \left[C_{ijlm}\varepsilon_{lm,y}\left(w\right)\right]}{\partial y_{j}}=\frac{\partial C_{ijkh}}{\partial y_{j}}\;,\;i=1,2,3$$

with the boundary conditions:

(44) $${\left.w^{kh}\right|}_{\partial \Gamma }=0$$

and the supplementary conditions:

(45) $$\langle w^{kh}\rangle =0$$

Let us take $\left(x_{1},x_{2},x_{3}\right)$ as the principal material axis, then, for a transversely isotropic material:

(46) $$C_{1111}=C_{11}\;C_{2222}=C_{22}\;C_{1122}=C_{1133}=C_{12}\;C_{2211}=C_{3311}=C_{21}\;C_{3322}=C_{2233}=C_{23}\;C_{3333}=C_{33}\;C_{4444}=\left(C_{22}-C_{23}\right)/2\;C_{5555}=C_{44}\;C_{6666}=C_{44}$$

and all other components of $C_{ijkl}$ are identically zero. Under the plain strain loading condition, following stress-strain relations can be written:

(47) $$\left\{\begin{array}{c} \sigma_{22}\\ \sigma_{33}\\ \tau_{23} \end{array}\right\}=\left[\begin{array}{ccc} C_{22} & C_{23} & 0\\ C_{23} & C_{33} & 0\\ 0 & 0 & \frac{C_{22}-C_{23}}{2} \end{array}\right]\left\{\begin{array}{c} \varepsilon_{22}\\ \varepsilon_{33}\\ \gamma_{23} \end{array}\right\}$$

or alternatively as:

(48) $$\left\{\sigma \right\}=\left[C\right]{\left\{\varepsilon \right\}}^{o}$$

The equilibrium conditions written previously become:

(49) $$\left[\begin{array}{ccc} \frac{\partial }{\partial y_{2}} & 0 & \frac{\partial }{\partial y_{3}}\\ 0 & \frac{\partial }{\partial y_{3}} & \frac{\partial }{\partial y_{2}} \end{array}\right]\left\{\begin{array}{c} \sigma_{22}\\ \sigma_{33}\\ \tau_{23} \end{array}\right\}=0$$

Or in a compact form:

(50) $$\left[\partial \right]\left\{\sigma \right\}=\left[\partial \right]\left[C\right]{\left\{\varepsilon \right\}}^{o}=0$$

The Equation (50) can then be written:

(51) $${\left\{\varepsilon \right\}}^{o}={\left\{\varepsilon \right\}}_{,x}^{u^{o}}+{\left\{\varepsilon \right\}}_{,y}^{u^{1}}$$

In this case, the equilibrium equations become:

(52) $$-\left[\begin{array}{ccc} \frac{\partial }{\partial y_{2}} & 0 & \frac{\partial }{\partial y_{3}}\\ 0 & \frac{\partial }{\partial y_{3}} & \frac{\partial }{\partial y_{2}} \end{array}\right]\left[\begin{array}{ccc} C_{22}^{\left(\lambda \right)} & C_{23}^{\left(\lambda \right)} & 0\\ C_{23}^{\left(\lambda \right)} & C_{33}^{\left(\lambda \right)} & 0\\ 0 & 0 & \frac{C_{22}^{\left(\lambda \right)}-C_{23}^{\left(\lambda \right)}}{2} \end{array}\right]{\left\{\varepsilon \right\}}_{,y}^{u^{1}}\;=\left[\begin{array}{ccc} \frac{\partial }{\partial y_{2}} & 0 & \frac{\partial }{\partial y_{3}}\\ 0 & \frac{\partial }{\partial y_{3}} & \frac{\partial }{\partial y_{2}} \end{array}\right]\left[\begin{array}{ccc} C_{22}^{\left(\lambda \right)} & C_{23}^{\left(\lambda \right)} & 0\\ C_{23}^{\left(\lambda \right)} & C_{33}^{\left(\lambda \right)} & 0\\ 0 & 0 & \frac{C_{22}^{\left(\lambda \right)}-C_{23}^{\left(\lambda \right)}}{2} \end{array}\right]{\left\{\varepsilon \right\}}_{,x}^{u^{o}}$$

The term ${\left\{\varepsilon \right\}}_{,x}^{u^{o}}$ does not depend on y. The coefficients $C_{ij}^{\left(\lambda \right)}$ are constants in the two phases. It should be mentioned again that in the above equations and those which follow, $\left(\lambda \right)$ represents both fiber $\left(\lambda =f\right)$ and matrix $\left(\lambda =m\right)$ constituents in the unit cell. In the case of plane strain loading conditions and using the matrix notation, it follows that:

(53) $$\left[\begin{array}{ccc} \frac{\partial }{\partial y_{2}} & 0 & \frac{\partial }{\partial y_{3}}\\ 0 & \frac{\partial }{\partial y_{3}} & \frac{\partial }{\partial y_{2}} \end{array}\right]\left[\begin{array}{ccc} C_{22}^{\left(\lambda \right)} & C_{23}^{\left(\lambda \right)} & 0\\ C_{23}^{\left(\lambda \right)} & C_{33}^{\left(\lambda \right)} & 0\\ 0 & 0 & \frac{C_{22}^{\left(\lambda \right)}-C_{23}^{\left(\lambda \right)}}{2} \end{array}\right]{\left\{\varepsilon \right\}}_{,y}^{u^{1}}=0$$

Now, when the functions $w^{kh}$ are determined for the above conditions, one may write:

(54) $${\left\{\varepsilon \right\}}_{,y}^{u^{1}}=\left[\begin{array}{ccc} \varepsilon_{22}\left(w^{22}\right) & \varepsilon_{22}\left(w^{33}\right) & \varepsilon_{22}\left(w^{23}\right)\\ \varepsilon_{33}\left(w^{22}\right) & \varepsilon_{33}\left(w^{33}\right) & \varepsilon_{33}\left(w^{23}\right)\\ \varepsilon_{23}\left(w^{22}\right) & \varepsilon_{23}\left(w^{33}\right) & \varepsilon_{23}\left(w^{23}\right) \end{array}\right]{\left\{\varepsilon \right\}}_{,x}^{u^{o}}$$

or, in an alternative form:

(55) $${\left\{\varepsilon \right\}}_{,y}^{u^{1}}=\left[\begin{array}{ccc} \left\{\varepsilon \left(w^{22}\right)\right\} & \left\{\varepsilon \left(w^{33}\right)\right\} & \left\{\varepsilon \left(w^{23}\right)\right\} \end{array}\right]{\left\{\varepsilon \right\}}_{,x}^{u^{o}}$$

Additionally:

(56) $$\left[\begin{array}{ccc} \frac{\partial }{\partial y_{2}} & 0 & \frac{\partial }{\partial y_{3}}\\ 0 & \frac{\partial }{\partial y_{3}} & \frac{\partial }{\partial y_{2}} \end{array}\right]\left[\begin{array}{ccc} C_{22}^{\left(\lambda \right)} & C_{23}^{\left(\lambda \right)} & 0\\ C_{23}^{\left(\lambda \right)} & C_{33}^{\left(\lambda \right)} & 0\\ 0 & 0 & \frac{C_{22}^{\left(\lambda \right)}-C_{23}^{\left(\lambda \right)}}{2} \end{array}\right]\left[\begin{array}{ccc} \left\{\varepsilon \left(w^{22}\right)\right\} & \left\{\varepsilon \left(w^{33}\right)\right\} & \left\{\varepsilon \left(w^{23}\right)\right\} \end{array}\right]{\left\{\varepsilon \right\}}_{,x}^{u^{o}}=0$$

(57) $$\left[\begin{array}{ccc} \left\{\varepsilon \left(w^{22}\right)\right\} & \left\{\varepsilon \left(w^{33}\right)\right\} & \left\{\varepsilon \left(w^{23}\right)\right\} \end{array}\right]{\left\{\varepsilon \right\}}_{,x}^{u^{o}}=0$$

This relation should remain valid for all ${\left\{\varepsilon \right\}}_{,x}^{u^{o}}$. With constant coefficients in both phases namely in the fiber and matrix, Equation (43) becomes:

(58) $$-\frac{C_{ijlm}^{\left(\lambda \right)}\partial \varepsilon_{lm,y}^{\left(\lambda \right)}(w^{kh})}{\partial y_{j}}=0\;,\;i=1,2,3$$

For plane strain i=2,3 and j=2,3, therefore:

(59) $$C_{22}^{\left(\lambda \right)}\frac{\partial \varepsilon_{22}\left(w^{kh}\right)}{\partial y_{2}}+C_{23}^{\left(\lambda \right)}\frac{\partial \varepsilon_{33}\left(w^{kh}\right)}{\partial y_{2}}+\frac{1}{2}\left[C_{22}^{\left(\lambda \right)}-C_{23}^{\left(\lambda \right)}\right]\frac{\partial \varepsilon_{23}\left(w^{kh}\right)}{\partial y_{3}}=0$$

and:

(60) $$C_{23}^{\left(\lambda \right)}\frac{\partial \varepsilon_{22}\left(w^{kh}\right)}{\partial y_{3}}+C_{22}^{\left(\lambda \right)}\frac{\partial \varepsilon_{33}\left(w^{kh}\right)}{\partial y_{3}}+\frac{1}{2}\left[C_{22}^{\left(\lambda \right)}-C_{23}^{\left(\lambda \right)}\right]\frac{\partial \varepsilon_{23}\left(w^{kh}\right)}{\partial y_{2}}=0$$

It should be noted that the above equations do not depend on k and j. This means that if one determines for example $w^{22}$, the remaining functions $w^{33}=w^{23}=w^{32}=w$ are immediately obtained. The solution of the above differential equation is composed of:

(61) $$w^{kh}=\left\{\begin{array}{c} w^{kh,(f)}\;for\;y\in V_{f}\\ w^{kh,(m)}\;for\;y\in V_{m} \end{array}\right.$$

which should satisfy the boundary conditions:

(62) $${\left.w^{kh,(f)}\right|}_{\partial \Gamma }=\;{\left.w^{kh,(m)}\right|}_{\partial \Gamma }$$

and:

(63) $$\sigma_{ij}^{\left(f\right)}n_{j}=\sigma_{ij}^{\left(m\right)}n_{j}$$

These correspond to the continuity conditions of the displacements and of the stresses at the boundary ∂Γ of the two phases.

The following problem will now be considered: Let the repeating periodic cell be subjected to the boundary condition: $u_{i}^{}=\alpha_{ij}y_{j}$. If the material is homogeneous, the average strain in the materials can be shown to be: $\langle \varepsilon_{ij}\rangle ={\bar{\varepsilon }}_{ij}=\alpha_{ij}$. This will be demonstrated later in the chapter. Now, let us solve the problem of the elasticity where a periodical cell is subjected to the condition indicated above. Furthermore, let us denote the displacement field by $w^{*}$ with the property ${\left.w^{*}\right|}_{\partial \Gamma }={\left.u\;\right|}_{\partial \Gamma }$ and ${\bar{\varepsilon }}_{kh}\left(w^{*}\right)=\alpha_{ij}$. Due to the existing symmetry in the distribution of the unit cell it can be concluded that $\langle w^{*}\rangle =0$. Let the field w also be introduced as:

(64) $$w=w^{*}-u$$

Corresponding to the conditions:

(65) $${\left.w\;\right|}_{\partial \Gamma }=0$$

and:

(66) $$\langle w\rangle =\langle w^{*}-u\rangle =\langle w^{*}\rangle -\langle u\rangle =0$$

This function $\left(w\right)$ verifies the condition of zero average and has the value zero on the contour and it is also a verification of Equation (60). For the “quasi-periodical fields” $u{\;}_{1}$, it follows that:

(67) $$u_{1}=\alpha_{22}\left\{\begin{array}{c} \frac{w_{22}^{*}}{\alpha_{22}}-y_{2}\\ 0 \end{array}\right\}+\alpha_{33}\left\{\begin{array}{c} 0\\ \frac{w_{22}^{*}}{\alpha_{22}}-y_{2} \end{array}\right\}$$

The strain fields for this displacement field are:

(68) $$\varepsilon_{22}\left(w^{22}\right)=\frac{\varepsilon_{22}\left(w^{*}\right)}{\alpha_{22}}-1\;;\;\varepsilon_{33}\left(w^{22}\right)=0$$

(69) $$\varepsilon_{22}\left(w^{33}\right)=\frac{\varepsilon_{22}\left(w^{*}\right)}{\alpha_{33}}-1\;;\;\varepsilon_{33}\left(w^{33}\right)=0$$

and:

(70) $$\varepsilon_{22}\left(u_{1}\right)=\varepsilon_{22}\left(w^{*}\right)-\alpha_{22}\;;\varepsilon_{33}\left(u_{1}\right)=\varepsilon_{33}\left(w^{*}\right)-\alpha_{33}\;$$

which can be written in a simplified form as:

(71) $${\bar{\varepsilon }}_{22}\left(w^{22}\right)=\frac{{\bar{\varepsilon }}_{22}\left(w^{*}\right)}{\alpha_{22}}-1=0\;;\;{\bar{\varepsilon }}_{33}\left(w^{33}\right)=\frac{{\bar{\varepsilon }}_{33}\left(w^{*}\right)}{\alpha_{33}}-1=0\;$$

Recall that for fiber reinforced unidirectional composite $C_{ijkh}^{\left(f\right)}$ and $C_{ijkh}^{\left(m\right)}$ represent the material coefficients for the fiber and matrix, respectively. The homogenized coefficients can be obtained using the following formula:

(72) $$C_{ijkh}^{o}=\langle C_{ijkh}\rangle +\langle C_{ijkh}\varepsilon_{lm,y}\left(w^{kh}\right)\rangle \;=\frac{1}{V}\int_{\Gamma }C_{ijkh}dV+\frac{1}{V}\int_{\Gamma }C_{ijkh}\varepsilon_{lm}\left(w^{*}\right)dV\;=\frac{1}{V}\left(C_{ijkh}^{\left(f\right)}V_{f}+C_{ijkh}^{\left(m\right)}V_{m}\right)+\frac{1}{V}\left(C_{ijlm}^{\left(f\right)}{\bar{\varepsilon }}_{lm}^{\left(f\right)}\left(w\right)V_{f}+C_{ijlm}^{\left(m\right)}{\bar{\varepsilon }}_{lm}^{\left(m\right)}\left(w\right)V_{m}\right)$$

with:

(73) $$\alpha_{lm}{\bar{\varepsilon }}_{lm}^{\left(f\right)}\left(w\right)={\bar{\varepsilon }}_{lm}^{\left(f\right)}\left(w^{*}\right)-\alpha_{lm}\;;\;\alpha_{lm}{\bar{\varepsilon }}_{lm}^{\left(m\right)}\left(w\right)={\bar{\varepsilon }}_{lm}^{\left(m\right)}\left(w^{*}\right)-\alpha_{lm}$$

Thus, one can show that:

(74) $$C_{ijkh}^{o}=v_{f}C_{ijkh}^{\left(f\right)}+v_{m}C_{ijkh}^{\left(m\right)}+v_{f}C_{ijkh}^{\left(f\right)}\left[\frac{{\bar{\varepsilon }}_{lm}^{\left(f\right)}\left(w*\right)}{\alpha_{lm}}-1\right]+v_{m}C_{ijkh}^{\left(m\right)}\left[\frac{{\bar{\varepsilon }}_{lm}^{\left(m\right)}\left(w*\right)}{\alpha_{lm}}-1\right]$$

and for the plane strain loading conditions, it yields:

(75) $$C_{22}^{o}=v_{f}C_{22}^{\left(f\right)}+v_{m}C_{22}^{\left(m\right)}+v_{f}C_{22}^{\left(f\right)}\left[\frac{{\bar{\varepsilon }}_{22}^{\left(f\right)}\left(w*\right)}{\alpha_{22}}-1\right]+v_{m}C_{22}^{\left(m\right)}\left[\frac{{\bar{\varepsilon }}_{22}^{\left(m\right)}\left(w*\right)}{\alpha_{22}}-1\right]\;=v_{f}C_{22}^{\left(f\right)}\frac{{\bar{\varepsilon }}_{22}^{\left(f\right)}\left(w*\right)}{\alpha_{22}}+v_{m}C_{22}^{\left(m\right)}\frac{{\bar{\varepsilon }}_{22}^{\left(m\right)}\left(w*\right)}{\alpha_{22}}$$

and:

(76) $$C_{23}^{o}=v_{f}C_{23}^{\left(f\right)}+v_{m}C_{23}^{\left(m\right)}+v_{f}C_{23}^{\left(f\right)}\left[\frac{{\bar{\varepsilon }}_{33}^{\left(f\right)}\left(w*\right)}{\alpha_{33}}-1\right]+v_{m}C_{23}^{\left(m\right)}\left[\frac{{\bar{\varepsilon }}_{33}^{\left(m\right)}\left(w*\right)}{\alpha_{33}}-1\right]\;=v_{f}C_{23}^{\left(f\right)}\frac{{\bar{\varepsilon }}_{33}^{\left(f\right)}\left(w*\right)}{\alpha_{33}}+v_{m}C_{23}^{\left(m\right)}\frac{{\bar{\varepsilon }}_{33}^{\left(m\right)}\left(w*\right)}{\alpha_{33}}$$

Now, in order to determine the coefficients $C_{ij}^{o}$ in the above equations, it is necessary to obtain the field of strain and finally compute the average values of strain in both phases of the composite. This is analytically feasible for very simple problems which are usually presented in literature. However, in the case of fiber reinforced composites, analytical computation of the strain field is a complex and tedious task. Nevertheless, by using the method of finite elements, evaluation of the field if strain and/or stress becomes a relatively easy process.

## 3. Experimental Creep Response of Fiber Reinforced Composite

If the engineering constants for a material have been determined, the creep behavior of the materials can be studied. The equipment belongs to the mechanical testing laboratory of the Technical University of Munich.

In order to verify the model proposed in the previous section, experiments on the behavior of carbon fiber reinforced composite have been made (Figure 1). The variable parameters are the stress acting on the test specimen and the temperature. The results are presented in Figure 2, Figure 3, Figure 4, Figure 5, Figure 6, Figure 7, Figure 8 and Figure 9.

To perform the experiment commercially available composites have been used. The epoxy material used is Fibredux 6376C, (manufactured by Novartis AG, Basel, Switzerland). This material is reinforced with carbon fibers T800 (made Toray International, Inc, Tokyo, Japan). The thermoplastic specimens was made by APC2 material. In this case the PEEK matrix (in a semicrystalline state) has a glass transition to a temperature of 145 °C. This material is reinforced with carbon fibers (Ashland Inc, Lexington, KY, USA) of the type IM6.

The dimensions of the specimens are: length of 150 mm, width of 10 mm and thickness of 1 mm. All the specimens, made by different materials have the same dimensions. The specimens have been stored in desiccants filled chamber in the period before testing (to protect the specimens from humidity—the relative humidity in this chamber was 35%).

## 4. Conclusions and Discussion

It has been shown in the paper that a very good approximation of the creep response of a material can be obtained if the homogenization theory is used to determine the mechanical constants of a fiber-reinforced composite material. In this way, the homogenization theory becomes a useful method for the study and determination of the mechanical properties of such a material. Inside the paper, two materials were studied; namely, Carbon/Epoxy and Carbon/PEEK at different temperatures and different loads. Each time very good values were obtained, close to the values obtained experimentally. Therefore, homogenization theory is proving to be a useful tool for carbon fiber composites.

The influence of temperature on the behavior of the material proves to be nonlinear. As a result, in practice, special attention must be paid to a good modeling of the mechanical behavior of the material. The present study solves the problem which involves unidirectional composites with elastic but transversal isotropic fibers. This is important when we use carbon fiber, that exhibit this property (in contrast to fiberglass).

As a conclusion, the homogenization method proves to be a very powerful and accurate tool in estimating the creep response of a composite material. The measurements proved that the theoretical results obtained using the homogenization method fit very well with the results obtained from some experimental measurements.

The use of the method in the mechanical identification of the material represents an effective tool for determining the time dependent overall response of any unidirectional composite under various loading conditions with reasonable accuracy.

## Figures and Tables

**Figure 1:**
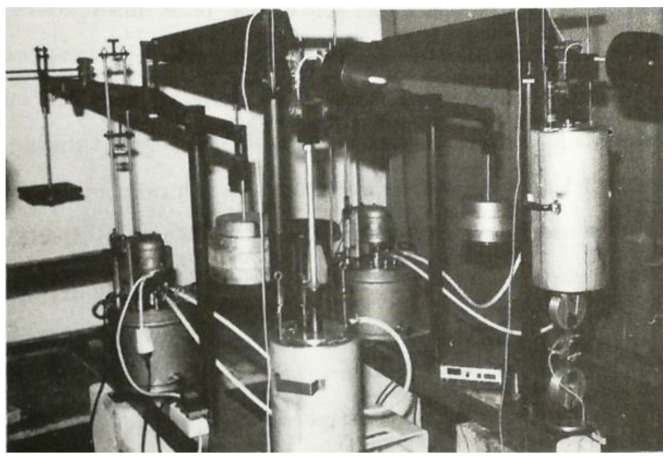
Experimental testing device.

**Figure 2:**
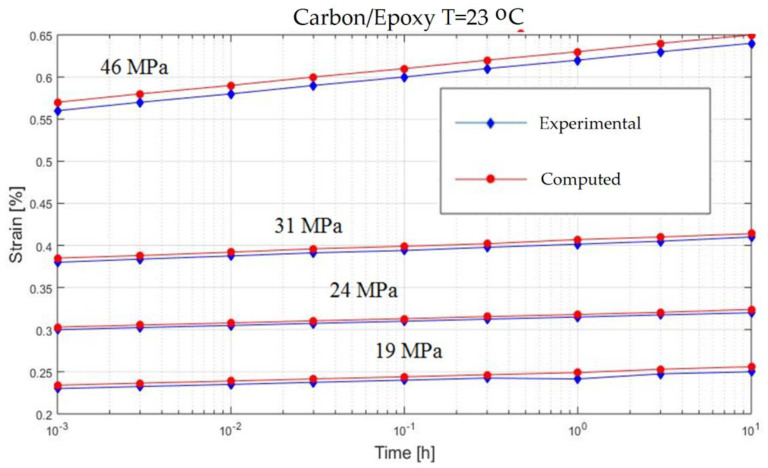
Creep response of a carbon/epoxy {90}_4s_ at T = 23 °C.

**Figure 3:**
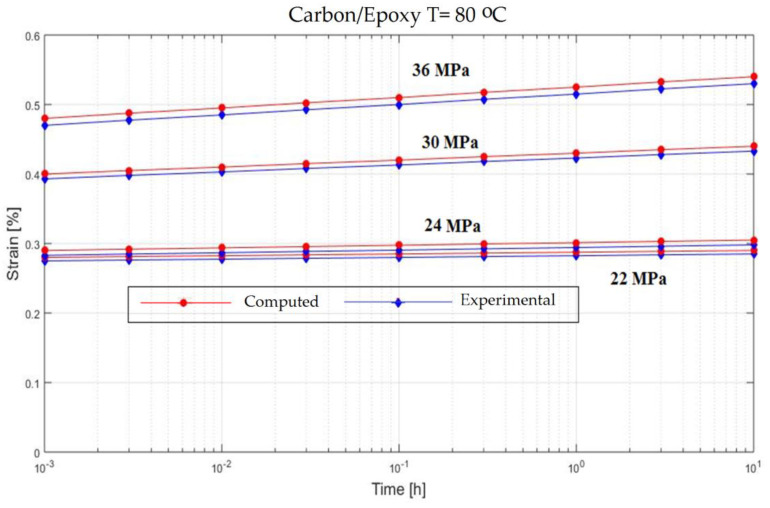
Creep response of a carbon/epoxy {90}_4s_ at T = 80 °C.

**Figure 4:**
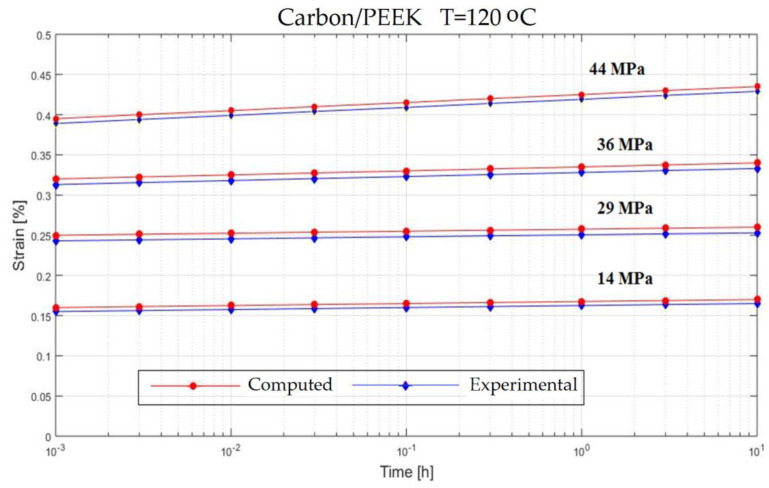
Creep response of a carbon/epoxy {90}_4s_ at T = 120 °C.

**Figure 5:**
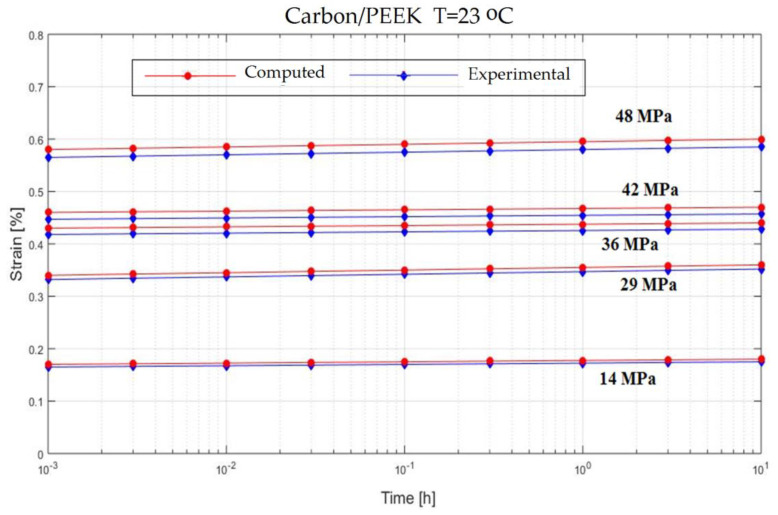
Creep response of a carbon/epoxy {90}_4s_ at T = 23 °C.

**Figure 6:**
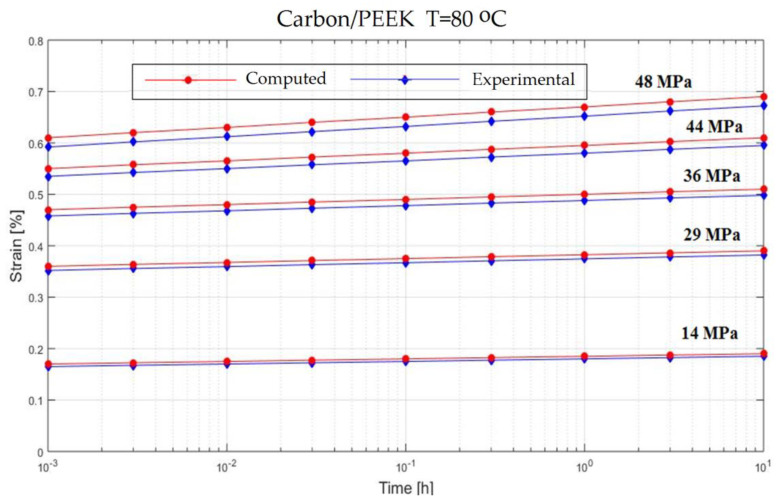
Creep response of a carbon/epoxy {90}_4s_ at T = 80 °C.

**Figure 7:**
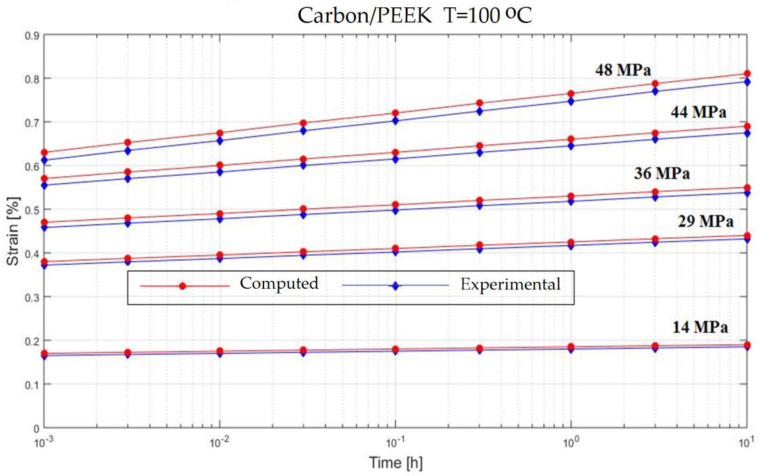
Creep response of a carbon/epoxy {90}_4s_ at T = 100 °C.

**Figure 8:**
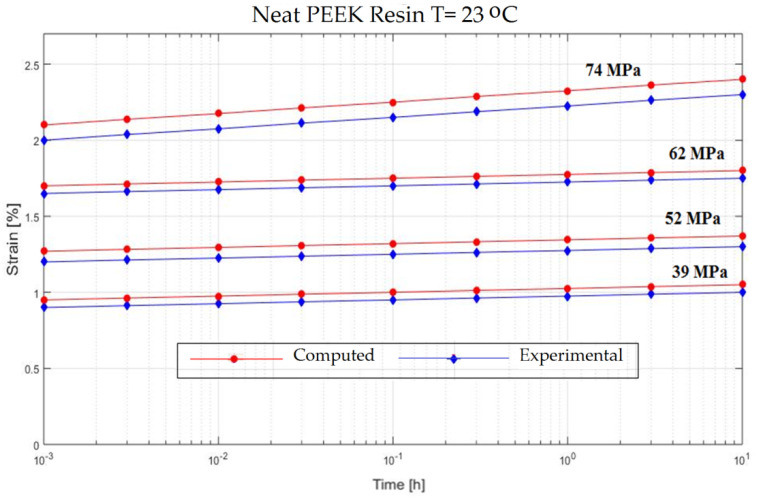
Creep response of a Neat PEEK Resin at T = 23 °C.

**Figure 9:**
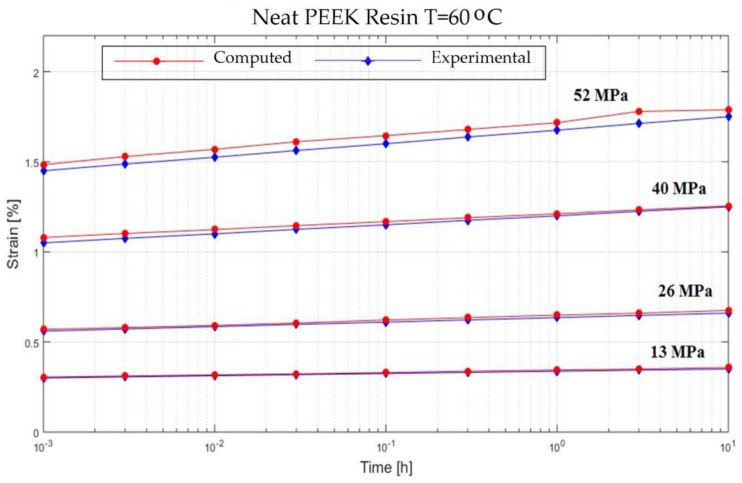
Creep response of a Neat PEEK Resin at T = 60 °C.

**Figure A1:**
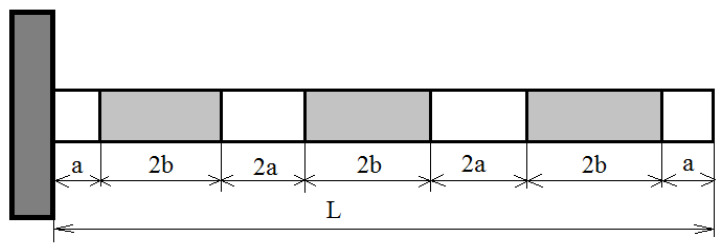
A one-dimensional theoretical composite.

## Data Availability

Not applicable.

## Appendix A

In this example (Figure A1), for notational simplicity, $\langle p\rangle$ will be replaced by $\bar{p}$ to imply the average, when p is a symbol representing the values of stresses and strains.

Let us consider a bar of length *L* loaded at its ends with $\bar{\sigma }$. The bar is made up of two materials with Young’s moduli $E_{a}$ and $E_{b}$ as shown in Figure A1. The strain in each of the constituting materials is constant and equal to:

(A1) $${\bar{\varepsilon }}_{a}=\frac{\bar{\sigma }}{E_{a}},\;{\bar{\varepsilon }}_{b}=\frac{\bar{\sigma }}{E_{b}}$$

If the bar is assumed to consist of n periodic cells, with the cell having the length 2 (*a* + *b*) where 2*a* is the length of the material with Young’s modulus $E_{a}$ and 2*b* is the length of the second material with Young’s modulus $E_{b}$, the total length is then *L* = 2 (*a* + *b*) *n*. The total displacement *u* at the end is therefor $u=n\left(u_{a}+u_{b}\right)$, where $u_{a}=2{\bar{\varepsilon }}_{a}a$ and $u_{b}=2{\bar{\varepsilon }}_{b}b$. Thus:

(A2) $$u=2n\left(a{\bar{\varepsilon }}_{a}+b{\bar{\varepsilon }}_{b}\right)=2n\bar{\sigma }\left(\frac{a}{E_{a}}+\frac{b}{E_{b}}\right)$$

The average strain is:

(A3) $$\bar{\varepsilon }=\frac{u}{L}=\frac{\bar{\sigma }}{a+b}\left(\frac{a}{E_{a}}+\frac{b}{E_{b}}\right)$$

By using $\bar{\varepsilon }=\bar{\sigma }/E$ the above equation can be simplified to:

(A4) $$\frac{a+b}{E}=\frac{a}{E_{a}}+\frac{b}{E_{b}}$$

where E referred to as the equivalent Young’s modulus.

Let at this point the homogenization theory be applied to this problem. The distribution of the displacement in the repeating unit cell is represented in Figure A1.

The relation between the strains in the two existing phases and the total average strain is:

(A5) $${\bar{\varepsilon }}_{a}^{*}=\frac{{\bar{\varepsilon }}_{a}}{\bar{\varepsilon }}=\frac{a+b}{E_{a}}\left(\frac{a}{E_{a}}+\frac{b}{E_{b}}\right);\;{\bar{\varepsilon }}_{b}^{*}=\frac{{\bar{\varepsilon }}_{b}}{\bar{\varepsilon }}=\frac{a+b}{E_{b}}\left(\frac{a}{E_{a}}+\frac{b}{E_{b}}\right)$$

where the Equations (44) and (45) have also been considered.

The formula for the Young’s modulus is:

(A6) $$E=\nu_{a}E_{a}+\nu_{b}E_{b}+\nu_{a}E_{a}\left({\bar{\varepsilon }}_{a}^{*}-1\right)+\nu_{b}E_{b}\left({\bar{\varepsilon }}_{b}^{*}-1\right){\bar{\varepsilon }}_{b}^{*}\;=\frac{a}{a+b}E_{a}+\frac{b}{a+b}E_{b}+\frac{a}{a+b}E_{a}\left[\frac{a+b}{E_{a}}/\left(\frac{a}{E_{a}}+\frac{b}{E_{b}}\right)-1\right]+\frac{b}{a+b}E_{b}\left[\frac{a+b}{E_{a}}/\left(\frac{a}{E_{a}}+\frac{b}{E_{b}}\right)-1\right]$$

Which can be simplified to:

(A7) $$E=\frac{a+b}{\frac{a}{E_{a}}+\frac{b}{E_{b}}}$$

The above example demonstrates that the method just presented can be applied analytically in a rather simple way, to a uni-dimensional case. For bi-dimensional problems however, the analytical approach becomes a difficult task and therefore, the problem has to be analyzed numerically.
